# Plant Biostimulants to Enhance Abiotic Stress Resilience in Crops

**DOI:** 10.3390/ijms26031129

**Published:** 2025-01-28

**Authors:** Luciana Di Sario, Patricia Boeri, José Tomás Matus, Gastón A. Pizzio

**Affiliations:** 1 CIT Río Negro, Universidad Nacional de Río Negro, Viedma CP8500, Río Negro, Argentina; ldisario@unrn.edu.ar (L.D.S.); pboeri@unrn.edu.ar (P.B.); 2 Institute for Integrative Systems Biology (I2SysBio), Universitat de València-CSIC, 46908 Paterna, Valencia, Spain; tomas.matus@uv.es

**Keywords:** crop resilience, sustainable agriculture, plant physiology, stress tolerance, hormonal regulation, antioxidant defense, metabolic adjustments, drought, salinity, temperature extremes, nutrient deficiencies, crop productivity, environmental challenges

## Abstract

The escalating impact of abiotic stress on crop productivity requires innovative strategies to ensure sustainable agriculture. This review examines the promising role of biostimulants in mitigating the adverse effects of abiotic stress on crops. Biostimulants, ranging from simple organic compounds to complex living microorganisms, have demonstrated significant potential in enhancing plant resilience, stress tolerance, and overall performance. The mechanisms underlying biostimulant action—such as enhancing antioxidant defenses, regulating hormonal pathways, and inducing metabolic adjustments—are reviewed. Furthermore, we incorporate the latest research findings, methodologies, and advancements in biostimulant applications for addressing abiotic stressors, including drought, salinity, high temperatures, and nutrient deficiencies. This review also highlights current challenges and future opportunities for optimizing biostimulant use in sustainable crop production. This revision aims to guide researchers and agronomists in applying biostimulants to improve crop resilience in the context of climate change.

## 1. Introduction

**Figure 1:**
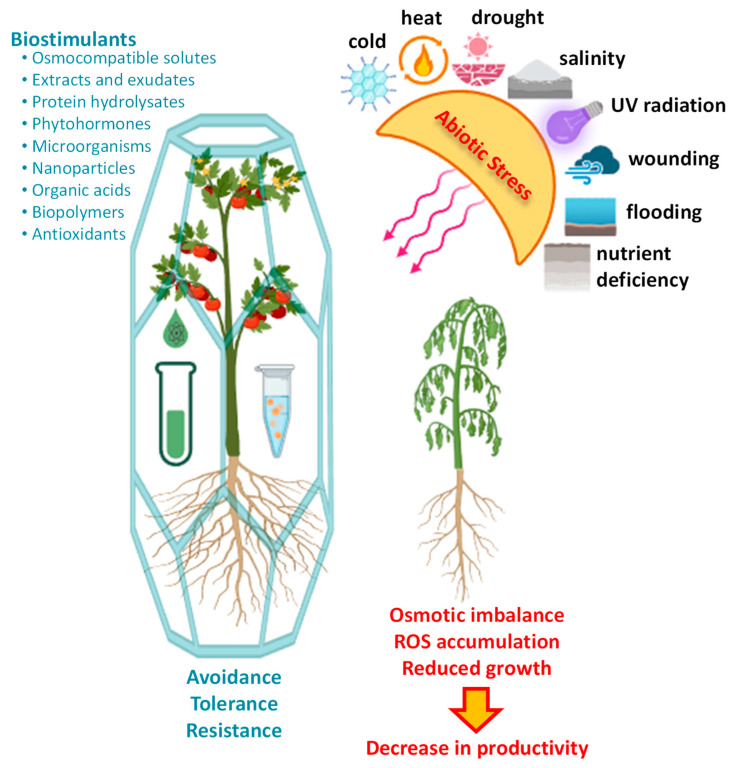
The role of biostimulants in mitigating the negative effects of abiotic stress on crop productivity. Abiotic stress conditions, such as drought, salinity, extreme temperatures, and nutrient deficiencies, induce detrimental changes at morphological, physiological, biochemical, and molecular levels, severely limiting plant growth and productivity. Biostimulants help crops counteract these stress-induced damages, restoring plant performance and enhancing resilience against environmental stressors. Illustration created using BioRender.

Agriculture and food production face unprecedented challenges in the 21st century, driven by climate change and the concomitant increase in abiotic stress on crop productivity [1]. Abiotic stressors such as drought, salinity, high temperatures, and nutrient deficiencies, among others, pose a significant threat to global food security (Figure 1). As climate change intensifies, the frequency and severity of these stressors continue to rise, requiring the development of innovative and sustainable agricultural practices [2]. In this context, the exploration of plant biostimulants as a strategic tool to enhance crop resilience against abiotic stress has garnered significant attention [3]. Biostimulants, comprising a diverse array of organic compounds derived from different sources (Figure 1), hold immense potential to elicit positive physiological responses in plants. These responses can enhance a plant’s capacity to withstand and recover from environmental challenges [4,5]. This comprehensive review provides an examination of the current state of knowledge regarding the use of plant biostimulants to mitigate abiotic stress in crops. Furthermore, this review emphasizes the potential of biostimulants to induce crop resilience under suboptimal environmental conditions and explores their impact on plant physiology, highlighting their promise as a critical tool for sustainable agriculture in the face of climate change.

## 2. Abiotic Stress in Crops: Challenges and Implications

Agricultural productivity is intricately linked to the complex interplay between crop physiology and environmental conditions. However, the intensification of abiotic stressors represents a significant threat to global food security [1]. Drought, salinity, extreme temperatures, and nutrient deficiencies— exacerbated by climate change—impose unprecedented challenges to crop yields and agricultural sustainability (Figure 1). Understanding the mechanisms of abiotic stress tolerance is crucial for developing effective strategies to mitigate the adverse effects of these stressors on plant growth and productivity.

Abiotic stress induces a wide range of damage at the tissue, cellular, and molecular levels, leading to altered growth patterns, reduced photosynthetic efficiency, and compromised reproductive success, among other abnormalities [6,7]. For instance, water scarcity triggers stomatal closure, which limits CO_2_ uptake and disrupts leaf temperature regulation. Similarly, salinity stress disturbs ion homeostasis, resulting in toxic ion accumulation and osmotic imbalances. These physiological disruptions have direct repercussions on crop yield and quality. Moreover, the economic implications of yield losses due to abiotic stress are profound, affecting farmers’ livelihoods and exacerbating global food insecurity [8]. Efforts to counteract the effects of abiotic stress often involve increased resource use, such as excessive irrigation or fertilizers, contributing to environmental degradation. This highlights the urgent need for sustainable and resilient agricultural practices [9]. Climate change is acting as a catalyst, intensifying the frequency and severity of extreme weather events, including droughts, floods, and heat waves [10]. Predictive models project a grim future for agriculture if adaptive strategies are not implemented to enhance crop resilience and ensure food security.

To address these challenges, it is critical to develop agricultural management strategies that increase crop resilience to abiotic stressors, optimize resource use efficiency, and minimize environmental impact. Biostimulants represent a promising solution, offering innovative pathways to enhance crop resilience and mitigate the detrimental consequences of abiotic stress on global food production [11].

## 3. Biostimulants to Tackle Abiotic Stress in Crops

Biostimulants represent a diverse group of substances that, when applied to plants, induce physiological and molecular changes, enhancing growth, development, and stress tolerance. These changes include mechanisms from osmotic adjustment to antioxidant defense activation that improve plant resilience under adverse conditions (Figure 1). It is important to distinguish biostimulants from biofertilizers: while biofertilizers primarily focus on nutrient supply, biostimulants exert a broader influence on plant physiology. The EU regulation defines a biostimulant as “a product that stimulates plant nutrition processes independently of the product’s nutrient content, with the sole aim of improving one or more of the following characteristics of the plant or the plant rhizosphere: (a) nutrient use efficiency; (b) tolerance to abiotic stress; (c) quality traits; or (d) availability of confined nutrients in the soil or rhizosphere” [12].

Biostimulants can be applied using three main approaches: (1) during seed priming to improve seed performance, (2) as foliar sprays directly onto plant shoots, or (3) as solid or liquid amendments applied to plant substrates, such as soil or hydroponic solutions. The classification of biostimulants encompasses a wide variety of compounds, including phytohormones, organic acids, plant extracts, and microbial-based formulations, among others. Understanding the diversity of these compounds is crucial for tailoring their application to specific crops and environmental conditions, thereby maximizing their effectiveness in enhancing plant performance under abiotic stress.

### 3.1. Osmocompatible Solutes (OCSs)

These small organic molecules accumulate within plant cells without causing cellular damage. They play a critical role in osmoregulation, maintaining cell volume and counteracting the effects of water stress by increasing water potential [13,14]. OCSs can also protect membranes and modulate signaling pathways, making them potential biostimulants for improving plant abiotic stress tolerance. OCSs accumulate in the cytosols and vacuoles of plant cells in response to osmotic stress, helping maintain cell volume and turgor pressure. This prevents plasmolysis and the shrinkage of cells due to water loss, which can damage cellular structures and disrupt physiological processes [15,16]. In addition, OCSs can modulate signaling pathways involved in stress response, such as abscisic acid (ABA) signaling, which can further enhance stress tolerance. There are a variety of OCSs found in plants, including sugars (i.e., sucrose, raffinose, and trehalose), polyols or sugar alcohols (such as sorbitol, mannitol, and glycerol), amino acids, and derivatives (from proline to betaines).

**Figure 2:**
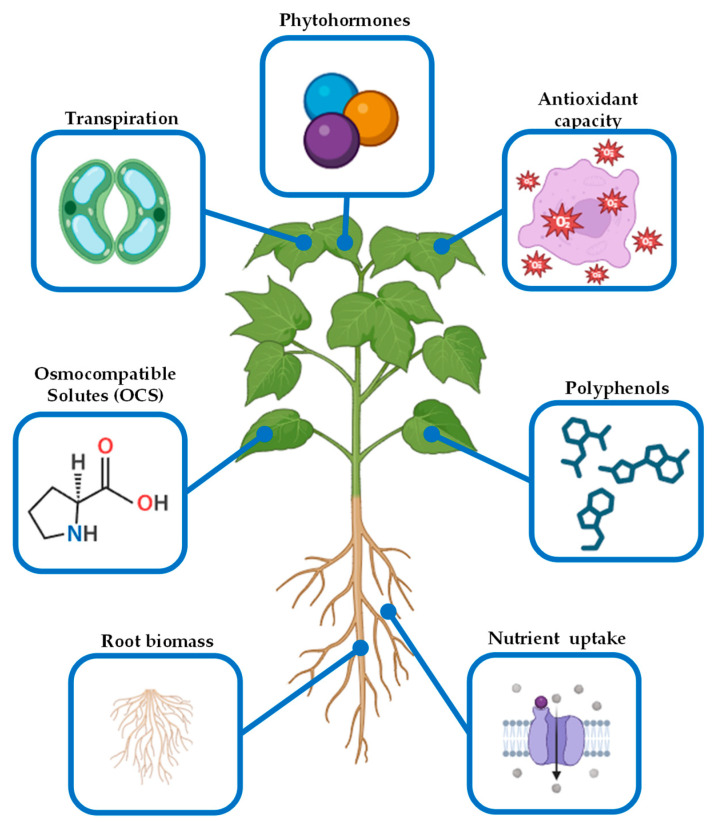
Molecular and physiological biostimulant-induced mechanisms to regulate abiotic stress tolerance in plants. Illustration created using BioRender.

OCSs have been shown to improve plant tolerance to a variety of abiotic stresses [17,18,19]. For instance, OCSs can enhance water uptake and reduce water loss through stomata, thereby improving drought tolerance. Moreover, OCSs can alleviate the negative effects of salt stress by reducing the osmotic potential of the soil solution and protecting cells from salt damage. In addition, OCSs can protect plants from heat and cold stress by maintaining membrane integrity and reducing the accumulation of reactive oxygen species (ROS) [20]. However, the effectiveness of OCSs as biostimulants depends on several factors, such as the type of stress, the plant species, and the formulation and application method, among others. On the other hand, OCSs offer several economic and environmental benefits as biostimulants. For instance, OCSs can enhance plant stress tolerance, reducing the need for chemical fertilizers and pesticides to protect against environmental stresses. In addition, OCSs can improve water and nutrient use efficiently, leading to increased crop yields, even under normal conditions. Moreover, they can reduce the environmental impact given that OCSs are typically derived from renewable resources and have a low environmental footprint [14,16,20]. OCSs are promising biostimulants with the potential to improve plant abiotic stress tolerance and enhance crop productivity (Figure 2). Their effectiveness and sustainability make them an attractive alternative to chemical inputs in modern agriculture. Further research is needed to optimize the use of OCSs as biostimulants and to develop novel formulations with enhanced properties. As our understanding of OCSs grows, their role in sustainable agriculture is likely to expand.

### 3.2. Antioxidants

Abiotic stress poses a significant challenge to modern agriculture, encompassing a spectrum of environmental factors such as drought, salinity, extreme temperatures, and heavy metal contamination that adversely affect plant growth and development. These stressors induce oxidative stress in plants through the excessive generation of reactive oxygen species (ROS), including singlet oxygen (^1^O_2_), superoxide radicals (O_2_−•), hydroxyl radicals (HO•), hydrogen peroxide (H_2_O_2_), alkoxyl radicals (RO•), and peroxyl radicals (ROO•). While ROS are essential for normal cellular signaling at basal levels, their overproduction under stress leads to oxidative damage, disrupting physiological processes and harming cellular components such as DNA, proteins, lipids, carbohydrates, and enzymes, ultimately triggering programmed cell death [21,22]. Oxidative damage also compromises membrane integrity and induces physiological and biochemical alterations that disrupt metabolism and reduce plant productivity [23,24]. Despite these challenges, plants have evolved endogenous mechanisms to combat oxidative stress. These mechanisms involve precise control of ROS levels through enzymatic and non-enzymatic antioxidant systems.

The enzymatic antioxidant defense system includes peroxidase (POD), superoxide dismutase (SOD), glutathione reductase (GR), catalase (CAT), dehydroascorbate reductase (DHAR), ascorbate peroxidase (APX), and monodehydroascorbate reductase (MDHAR). Non-enzymatic antioxidants include ascorbate (AsA), flavonoids, carotenoids, stilbenes, tocopherols, and other vitamins. These systems work collectively to detoxify excessive ROS and restore antioxidant homeostasis, thereby enhancing plant resilience to abiotic stress [25,26,27].

Antioxidants have emerged as promising biostimulants for mitigating the effects of abiotic stress. When applied exogenously, antioxidants perform multiple roles to alleviate the adverse effects of abiotic stress [28,29], including ROS scavenging and minimizing oxidative damage to cellular structures and biomolecules such as proteins, lipids, and nucleic acids. Moreover, antioxidants also regulate stress-signaling pathways, modulating the expression of genes involved in stress adaptation and defense mechanisms. They enhance photosynthetic efficiency, ensuring plants have sufficient energy to cope with adverse conditions. Several antioxidants have shown promise as biostimulants, including polyphenols, a diverse group of compounds found in plants, which include flavonoids, tannins, and stilbenes; vitamins, particularly vitamins C and E, which protect membranes by scavenging ROS and maintain cellular redox balance; phytosterols, compounds with structural roles in cell membranes and antioxidant activity; and Coenzyme Q10, an essential cofactor in the electron transport chain that also acts as a potential antioxidant [28,29,30,31].

The application of antioxidants as biostimulants presents a proven strategy for enhancing plant abiotic stress tolerance (Figure 2). However, further research is needed to fully elucidate their mechanisms of action under stress conditions, optimize formulations and application methods, and explore synergistic interactions with other compounds. Additionally, identifying novel antioxidant molecules through the screening of plant extracts or synthetic compounds with enhanced stress-protective properties offers a promising avenue for future research. The ability of antioxidants to mitigate ROS-induced damage, regulate stress signaling, and enhance stress adaptation highlights their value as tools for optimizing crop resilience and productivity.

### 3.3. Phytohormones

Plant hormones are critical signaling molecules that regulate plant metabolism, growth, development, and stress responses. Naturally produced by plants, they act in a coordinated manner to enable adaptation to environmental challenges. Phytohormones have gained attention as promising biostimulants for improving plant tolerance to abiotic stress [32,33,34]. Among them, abscisic acid (ABA) plays a pivotal role in regulating drought, salt, and cold stress responses by mediating stomatal closure and improving water use efficiency [35]. Similarly, salicylic acid (SA) contributes to plant defense mechanisms against biotic and abiotic stresses [36]. Gibberellins (GAs) are involved in promoting stress-induced growth responses and enhancing antioxidant defense [37]. Cytokinins can promote root growth and nutrient uptake under stress conditions [38] or auxins can regulate stomatal opening, nutrient uptake, and stress signaling pathways [39].

Phytohormones can alleviate the negative effects of abiotic stresses through different mechanisms, for instance, regulating stomatal opening and water use efficiency or protecting membranes and cellular structures through the enhancement of the antioxidant defense (Figure 2). In addition, phytohormones are able to promote nutrient uptake and metabolism to stimulate growth, enabling plants to better access essential resources under nutrient deficiency and environmental stress [32,33,34]. Considered one of the first studied biostimulants, their effectiveness can be influenced by factors such as their formulation and application method. Organic carriers and advanced technologies like nano-encapsulation have been developed to improve the delivery and efficiency of phytohormones. Application methods, including foliar sprays and soil treatments, provide flexible strategies to target specific crop requirements and environmental challenges. Ongoing research in this field is focused on developing innovative formulations and application techniques such as precision agriculture and controlled-release systems. Scientists are also investigating the synergistic effects of phytohormones with other biostimulants and nutrient management practices. Furthermore, long-term studies are being conducted to understand the impact of phytohormones on crop health and soil microbiomes, ensuring their sustainable use.

### 3.4. Extracts, Exudates and Protein Hydrolysates

Protein hydrolysates consist of a diverse combination of amino acids, oligopeptides, and soluble polypeptides, contingent upon the protein source and the applied processing methodologies [40]. These compounds serve as signaling molecules [41,42] and enter the plant cell through both diffusion processes as well as active transport that involve an energy cost [43]. In addition, applied to plants, protein hydrolysates alleviate osmotic stress by modulating primary and secondary metabolism [44]. The exogenous application of these products stimulates different physiological and molecular processes, enhancing nutrient absorption and utilization efficiency. Additionally, whether directly or indirectly, these applications mitigate the adverse effects of both abiotic and biotic stresses, ultimately enhancing crop yield and quality [43,45,46,47,48]. Concerning the mechanism of action of protein hydrolysates, studies conducted in lettuce and corn plants suggest that these biocompounds can positively regulate the expression levels of phenylalanine ammonium lyase (PAL). Consequently, they induce the production of secondary metabolites, including flavonoids, terpenes, carbohydrates, sterols, and amino acids, enhancing tolerance to abiotic stress [4]. Nevertheless, despite recent advances in understanding the mechanisms of action of biostimulants, studies aimed at elucidating their bioprotective effects against abiotic stresses remain limited. In relation to this, Paul et al. [49] pointed out that different protein hydrolysates applied to tomato plants could regulate ROS-mediated signaling, inducing changes in the levels of compounds with antioxidant activity, such as phenols and carotenoids. In addition, biostimulants based on seaweed extracts include a wide variety of bioactive compounds, from nutrients to phytohormones [50,51]. Although the mechanism by which these extracts enhance stress tolerance is not fully understood, regulatory molecules, osmoprotectants, transporters, and detoxifying enzymes have been reported to play a role [52]. For instance, betaines and cytokinins are components accountable for stress regulation [53,54]. Moreover, other compounds present in these extracts can act as signaling molecules that regulate key pathways at the transcriptional and/or post-translational level [55]. These molecules, in conjunction with polysaccharides, may undergo an endogenous increase in the presence of algal extracts [56]. The diversity of substances found in protein hydrolysates and seaweed extracts adds complexity to the comprehension of their mechanisms of action as biostimulants. In this context, numerous studies have highlighted the efficacy of these biocompounds in enhancing plant tolerance to both biotic and abiotic stressors across various agronomically significant species, including soybean (*Glycine max*) [57], rice (*Oryza sativa*) [58], wheat (*Triticum aestivum*) [59], tomato (*Solanum lycopersicon*) [60], arugula (*Eruca vesicaria*) [61], spinach (*Spinacia oleracea*) [62], squash (*Cucurbita pepo*) [63], peas (*Cajanus cajan*) [64], cucumber (*Cucumis sativus*) [65], okra (*Abelmoschus esculentus*), and cassava (*Manihot esculentus*) [66].

These findings not only expand our understanding of the impact of such biostimulants on plant physiology but also offer promising perspectives for enhancing crop productivity and quality in suboptimal environments. Thus, biostimulants derived from protein hydrolysates have the potential to positively transform modern agriculture, providing sustainable and effective solutions to address future challenges.

## 4. Plant-Derived Biostimulants

Plant-derived biostimulants comprise a heterogeneous set of products able to enhance crop production, impacting flowering, fruit development, root biomass, and responses to abiotic stress. Plant-derived biostimulants include different preparations such as phytohormones, specialized metabolites, extracts, and hydrolysates from whole plants, specific organs, cell cultures, and even plant by-products [67]. Furthermore, the different origins of the plant material used and the methods of preparation and application on crops add complexity to the biostimulant mechanisms of action and their effect on plant physiology. In this regard, the biostimulant action on plants may include mechanisms from hormonal effects [68] to their antioxidant activity [69] (Figure 2).

The phytohormone abscisic acid (ABA) is a key player in plant abiotic stress tolerance induction. For instance, ABA modulates plant transpiration via stomatal regulation under drought and heat stress in wheat (*Triticum aestivum*) [70]. In addition, it was shown that ABA signaling regulates the balance between transpiration and photosynthesis in *Nicotiana benthamiana*, regulating growth and drought stress tolerance [71] (Table 1). Moreover, ABA is capable of triggering osmotic adjustments, inducing proline and galactinol synthesis; galactinol is a precursor of the osmoprotective oligosaccharide raffinose family. Additionally, ABA regulates ripening, sugar accumulation, and color development in *Vitis vinifera* fruits [72]. Furthermore, ABA induces the production of anthocyanins and phenolic content, enhancing the plant’s antioxidant capacity [73,74]. Thereby, biostimulants formulated from plant tissues rich in ABA content, such as avocados, citrus, soybean, and figs [75], represent a sustainable strategy for agriculture to cope with abiotic stress; such formulations also have the potential to have a direct positive impact in human health [76]. Nevertheless, synthetic small chemicals with ABA receptor agonist activity, such as iSB09 and AMF4, are emerging as an improved alternative to ABA [77].

Melatonin (MEL) is another multifunctional growth regulator with the advantage of having direct antioxidant capacity (Figure 2). MEL is involved in different physiological processes from growth (i.e., promoting cell division and elongation, biomass accumulation, and meristematic growth), development (i.e., accelerating seed germination and retarding senescence), and stress response (i.e., enhancing drought, salinity, and heat stress tolerance) [78]. For instance, exogenous MEL application improves the germination rate of cotton (*Gossypium hirsutum*) seed by increasing antioxidant capacity and reducing ABA levels [79] (Table 1). Moreover, MEL-treated stevia (*Stevia rebaudiana*) seeds showed increased levels of phenolic compounds, an improvement in germination rate, higher plantlet fresh weight, and more leaves [80]. MEL treatments also enhance root growth and nutrient uptake in crops such as tomato (*Solanum lycopersicum*) [81], cucumber (*Cucumis sativus*) [82], and soybean (*Glycine max*) [83] (Table 1). On the other hand, MEL is able to enhance plant resilience to abiotic stress. For instance, MEL treatment on wheat (*Triticum aestivum*) improved the drought resistance of Chinese Spring, Shi4185, and Hanxuan10 varieties [84]. The molecular mechanism of this phenotype involves a decrease in drought-induced cell membrane damage through a reduction in hydrogen peroxide levels and an increase in jasmonic acid (JA) content by the transcriptomic regulation of LOX1.5 and LOX2.1 genes (involved in JA synthesis) and transcription factors such as HY5 and MYB86. Moreover, the resveratrol-rich medicinal plant *Polygonum cuspidatum* is a drought-sensitive crop. Exogenous MEL application is not only able to induce stress tolerances in *P. cuspidatum* but also increase resveratrol levels through the induction of stilbene-synthetic gene expression, boosting the antioxidant capacity [85]. Additionally, MEL-induced drought stress tolerance in maize (*Zea mays*) [86] and rice (*Oryza sativa*) [87] through the antioxidant defense system enhancement was also reported. On the other hand, MEL treatment on kiwifruit (*Actinidia chinensis*) plants diminishes oxidative damage induced by flooding, showing reduced ROS accumulation in roots [88] (Table 1). A similar phenotype was also described in wheat (*Triticum aestivum*), in which MEL-treated plants, subjected to flooding, showed an increase in antioxidant enzyme levels and a concomitant reduction in oxidative harm, improving wheat flooding tolerance [89].

Different authors have pointed out the key role of these bio-compounds in enhancing nutrient use efficiency [90]. Moreover, formulations based on protein hydrolysates are widely used due to their ability to enhance nutrient uptake, promote plant growth, and improve stress tolerance [91] (Figure 2). For instance, nitrogen (N) is a key nutrient for plant growth; it is a building block for amino acid and nucleotide synthesis. In this regard, the application of Trainer, a commercial legume-derived plant hydrolysate, increases N content in ornamental crops, such as *Begonia tuberhybrida*, *Pelargonium peltatum,* and *Viola cornuta*, enhancing plant growth and ornamental quality [92]. Trainer-induced nitrogen use efficiency increments were also shown in *Spinacia oleracea* and *Valerianella locusta* [93] (Table 1), with concomitant crop yield enhancement. Additionally, nutrient use efficiency and root growth in *Cannabis sativa* can be enhanced by a biostimulant complex composed of Aloe vera extract, fish hydrolysate, and kelp [94].

Plant-derived biostimulants also influence the synthesis of secondary metabolites, which play a crucial role in plant defense mechanisms against stress. These compounds, which include alkaloids, phenylpropanoids, terpenoids, and phenolic compounds, are essential for enhancing plant resistance to adverse conditions, such as biotic and abiotic stresses [95,96,97]. Furthermore, several studies have indicated that the exogenous application of plant extracts increases the polyphenol content in crops; for instance, the application of oak extracts in *Vitis vinifera* [98], moringa extracts in *Coriandrum sativum* [99], and alfalfa and red grape extracts in *Capsicum chinensis* [100] (Table 1). Additionally, grapevine treatment with vine-shoot extracts leads to higher terpene and norisoprenoid levels in fruits [101,102]. The application of *Callicarpa macrophylla* extracts, which are rich in Calliterpenone, increases the concentration of menthol in wild mint (*Mentha Arvensis*) [103] and the spry of moringa (*Moringa oleifera*) leaf extract on rose-scented geranium (Pelargonium graveolens), enhances geraniol, linalool, citronellol, and β-caryophyllene synthesis [104].

Understanding the modes of action of biostimulants requires integrating omics technologies, chemical bioprospecting, and mathematical tools, which enable the identification and characterization of active compounds and the detailed analysis of data. This comprehensive perspective is crucial for developing precise and optimized formulations tailored to each crop type [105], thus ensuring more effective and sustainable agricultural practices.

**Table 1:** Biostimulant effects on crops.

Biostimulant Source	Biostimulant	Treated Crop	Improvement in	Reference
	Abscisic acid	Nicotiana benthamiana	Drought stress tolerance	[71]
		Vitis vinifera	Ripening and fruit quality	[72]
			Antioxidant capacity	[73,74]
	Melatonin	Gossypium hirsutum	Germination/antioxidant capacity	[79]
		Stevia rebaudiana		[80]
		Solanum lycopersicum	Root growth/nutrient use efficiency	[81]
		Cucumis sativus		[82]
		Glycine max		[83]
		Triticum aestivum	Drought stress tolerance	[84]
		Polygonum cuspidatum	Drought stress tolerance/resveratrol levels	[85]
		Zea mays	Drought stress tolerance	[86]
		Oryza sativa		[87]
		Actinidia chinensis	Flood stress tolerance	[88]
		Triticum aestivum	Flood stress tolerance/antioxidant capacity	[89]
	Protein hydrolysates	Begonia tuberhybrida	Nutrient use efficiency/plant growth	[92]
		Pelargonium peltatum		
		Viola cornuta		
		Spinacia oleracea		[93]
		Valerianella locusta		
		Cannabis sativa		[94]
	Extracts	Vitis vinifera	Polyphenol content	[98]
		Coriandrum sativum		[99]
		Capsicum chinensis		[100]
		Vitis vinifera	Terpene and norisoprenoid content	[101,102]
		Mentha Arvensis	Menthol content	[103]
		Pelargonium graveolens	Geraniol, linalool, and citronellol content	[104]
Seaweeds	Fucans	Nicotiana tabacum	Biotic stress tolerance	[106]
	Carrageenans	Zea mays/Cicer arietinum	Plant growth	[107]
		Nicotiana tabacum		[108]
	Alginates	Foeniculum vulgare	Plant growth and development	[109]
	Carrageenans	Pinus radiata	Plant growth	[110]
		Eucalyptus globulus		[111]
	Alginates	Papaver somniferum	Plant growth and development	[112]
		Oryza sativa		[113]
		Arachis hypogaea		
		Triticum aestivum	Drought stress tolerance	[114]
	A. nodosum extract	Solanum lycopersicum	Heat stress tolerance	[115]
	G. rugosa extract		Drought stress tolerance	[116]
Microalgae	*Nannochloris* sp. extract	Solanum lycopersicum	Drought stress tolerance	[117]
	Spirulina platensis	Carica papaya	Plant growth	[118]
		Solanum melongena		[119]
	*A. platensis* and *Scenedesmus* sp.	Petunia hybrida		[120]
	A. fusiformis	Allium sativum		[121]
	Spirulina platensis	Capsicum annuum	Fruit yield and quality	[122]
Bacteria	PGPR	Phoenix dactylifera	Salt stress tolerance	[123]
		Oryza sativa	Salt stress tolerance	[124]
		Amaranthus viridis	Salt stress tolerance	[125]
		Hordeum vulgare	Drought stress tolerance	[126]
		Zea mays	Salt stress tolerance	[127]
		Solanum lycopersicon	Plant growth/fruit yield	[128]
	PSB	Zea mays	Nutrient use efficiency/salt tolerance	[129]
		Quercus Brantii	Drought stress tolerance	[130]
		Arachis hypogaea	Salt stress tolerance	[131]
		Solanum tuberosum	Plant growth	[132]
		Lycopersicon esculentum	Drought stress tolerance	[133]

## 5. Biostimulants from Seaweeds (Macroalgae) and Microalgae

Seaweeds, also known as marine macroalgae, are photosynthetic organisms that inhabit marine environments. They are classified as eukaryotic organisms, meaning that their cells contain a true nucleus and other membrane-bound organelles. Seaweeds comprise a group of macroscopic organisms that range in size from 0.5 mm and to 200 feet in length. These macroalgae constitute a significant category for the market of organic plant biostimulants [134]. They exhibit a complex and dynamic taxonomy that allows them to be classified, according to their pigmentation, into red algae (*Rhodophyta*, approximately 7500 species), brown algae (*Phaeophyta*, approximately 2000 species), and green algae (*Chlorophyta*, ~1500 algae) [135,136]. The marine environment they inhabit undergoes a series of constant modifications caused by tidal waves, changes in temperature, evaporation, precipitation, freshwater inflows, and sea level changes [137].

On the other hand, while salinity remains relatively constant in the open ocean, in semi-enclosed conditions, coastal areas, and estuaries, salinity changes are accentuated [138]. Intertidal environments represent transitional areas subjected to abrupt changes and recurrent fluctuations in environmental conditions, including intense radiation, high temperatures, desiccation, and salinity with changing tides, compounded by seasonal meteorological variations [137,139]. In these salt stress situations, algal cells continue to be in contact with water despite having a reduced water potential, while considerable cellular dehydration occurs as a result of desiccation. Both salinity and desiccation constitute two forms of water deprivation, for which the concept of “physiological drought” has been suggested [140]. The stress induced by these conditions causes a loss of water, ions, and electrolytes in the cell membrane, as well as pH modifications, crystallization of solutes, and denaturation of proteins. These events trigger the accumulation of reactive oxygen species (ROS) that modifies the redox homeostasis of the cell, causing an ‘oxidative stress’ that induces damage to the photosynthetic apparatus, DNA, proteins, and cell membranes [141,142]. However, ROS also acts as signaling molecules for cellular processes, including environmental stress tolerance. Therefore, cells must closely control ROS levels to avoid oxidative damage but allow signaling and tolerance induction [137].

Intertidal algae employ a diverse array of biochemical and physiological mechanisms to regulate homeostasis and sustain cellular integrity in suboptimal environments [143]. As a consequence, they synthesize a myriad of organic compounds, many of which have been recognized for their positive biostimulant effects on plants, including photosynthetic pigments, such as chlorophylls, carotenoids, and phycobiliproteins [144,145]. In addition, macro- and micronutrients are also found in seaweed products in fresh, dried, or extract forms [146]. On the other hand, among the secondary metabolites implicated in stress responses are polyphenolic compounds, including florotanins, bromophenols, flavonoids, phenolic terpenoids, mycosporine-like amino acids, and halogenated compounds [147,148].

In recent decades, more than 3000 compounds from macroalgae have been described, with recognized applications in the pharmaceutical, cosmetic, agricultural, bioenergy, and food sectors [149,150]. Many of these compounds have been linked to biostimulant effects on photosynthetic activities, nutrient uptake, and polyphenol accumulation, resulting in benefits for growth, resistance, fruit coloration, nutritional composition, and crop quality [55,151]. Thus, it has been demonstrated that most of the polysaccharides and their derived oligosaccharides activate defense and protection responses against a wide range of pathogens in terrestrial plants. Moreover, these compounds vary according to the type of algae used as raw material, with ulvans found in green algae; agarans and carrageenans in red algae; and alginates, fucans, and laminarin in brown algae [152,153]. It has been reported that these alginates and their oligoderivatives can trigger an initial burst of oxidation and activate signaling pathways that induce local and systemic defense responses in plants [106], leading to increased expression of defensive enzymes, such as phenylalanine synthase, ammonium lyase, and lipoxygenase. In turn, these compounds are involved in the synthesis of phenylpropanoids, terpenes, terpenoids, and alkaloids, which also exhibit antimicrobial activities [55,154], as well as stimulants of plant growth, development, and resistance [56,155]. Several investigations have demonstrated the biostimulant effect of these polysaccharides and derived oligosaccharides on agriculturally relevant crops. For instance, carrageenans and oligo-carrageenans showed plant growth enhancement in *Zea mays* and *Cicer arietinum* [107]. Similarly, these compounds promoted photosynthate, basal metabolism, and growth in tobacco (*Nicotiana tabacum* var. *burley*) [108], fennel (*Foeniculum vulgare*) [109], pine (*Pinus radiata*), and eucalyptus (*Eucalyptus globulus*) plants [110,111]. Additionally, a positive effect of alginates on crop growth was shown in poppy (*Papaver somniferum*) [112], rice (*Oryza sativa* var. *japonica*), and peanut (*Arachis hypogea*) [113], as well as drought stress tolerance induction in wheat (*Triticum aestivum*) [114] (Table 1; Figure 2). In addition, carbohydrate-rich *Ascophyllum nodosum* extracts have been reported to induce heat stress tolerance in tomato (*Solanum lycopersicum*) [115]. Furthermore, *Galaxaura rugosa* extracts applied to tomato roots induce drought tolerance [116]. The mechanism of this induction involves activating the abscisic acid (ABA) signaling pathway, leading to improved CO_2_ assimilation and water use efficiency. On the other hand, sulfated fucan oligosaccharides induced tolerance against tobacco mosaic virus in *Nicotiana tabacum* [106].

Currently, algae-derived products are one of the most promising and rapidly expanding categories in the biostimulant industry [115,156]. Their application has notable benefits for the agricultural sector, including the efficacy of these products at low concentrations and their ability to enhance the defensive responses of crops under abiotic stress conditions. Additionally, it has been reported that these biostimulants can improve crop yield and protein accumulation [155]. However, the formulation of new biostimulants presents certain difficulties, for instance the variability of the algae used as raw material [157,158]. Moreover, algae are exposed to a wide range of abiotic and biotic factors, such as species, seasonal changes, life cycle, size, age, reproductive status, location, depth, nutrients, salinity, light intensity, ultraviolet radiation, herbivory, and specific harvest times. Finally, some authors have warned about algae’s capacity to absorb heavy metals from their environments and bioaccumulate them [159]. Thus, sustained use of these compounds as crop biostimulants could result in the biomagnification of these heavy metals over time. Although these aspects represent significant disadvantages for the industry, algal biostimulants could also be used for the bioremediation of contaminated soils through biosorption processes [160]. Therefore, further research is necessary to help clarify these aspects and drive the advancement of the biostimulant industry. Moreover, seaweeds as plant biostimulants have a wide range of economic and potential applications, and their importance is likely to grow in the future.

Microalgae are photosynthetic single-cell microscopic organisms that are among the most ancient and diverse life forms on Earth. They inhabit a wide range of environments, including freshwater, saltwater, and extreme habitats such as deserts and hot springs. These microorganisms are characterized by their rapid reproduction rates, which make them an exceptionally productive source of biomass. Microalgae are classified based on different criteria, including pigmentation, life cycle, cell structure, and morphology. The group comprises both prokaryotic cyanobacteria, belonging to the divisions *Cyanophyta* and *Prochlorophyta*, and eukaryotic protists, which include *Glaucophyta*, *Rhodophyta*, *Heterokontophyta*, *Haptophyta*, *Cryptophyta*, *Dinophyta*, *Euglenophyta*, *Chlorarachniophyta*, and *Chlorophyta* [161,162].

The cultivation of microalgae, often referred to as unicellular biofactories, has gained attention for its beneficial effects in agriculture, particularly in promoting plant growth and enhancing plant responses to abiotic stress conditions [163,164,165,166,167,168]. One of the advantages of microalgae cultivation is its relative ease and cost-effectiveness [134]. Given their promising commercial potential as biostimulants and biofertilizers, numerous systems for microalgae biomass production have been developed, ranging from laboratory to industrial scales. Common methods include open pond or racetrack systems, both of which have been extensively studied and optimized [134,169,170].

Despite the increasing relevance of microalgae cultivation in agriculture, the market for microalgae remains less established compared to that of macroalgae [171]. However, their versatility and wide range of applications suggest significant growth potential for this emerging sector. The bioactive compounds in microalgae are contained within their cell walls and/or bound to specific cellular structures [172]. To access these compounds, it is necessary to employ different extraction processes, including enzymatic treatments that break down the cell walls and release their contents [117,173,174]. The vast species diversity of microalgae, combined with their unique biochemical compositions and adaptability to diverse environments, provides them with a broad spectrum of potential applications. Microalgae-based biostimulants have garnered significant interest as a source of macromolecules such as proteins, carbohydrates, and lipids, as well as high value-added products that can be extracted from their biomass, such as pigments, polyunsaturated fatty acids, peptides, exopolysaccharides, and amino acids, among others [175]. These bioactive compounds produced by microalgae are often associated with exceptionally high market values [161]. Notable species recognized for their biostimulant effects include *Chlorella vulgaris*, *Acutodesmus dimorphus*, *Scenedesmus platensis*, *Scenedesmus quadricauda*, *Dunaliella salina*, *Chlorella ellipsoida*, *Chlorella infusionum*, *Spirulina maxima,* and *Calothrix elenkinii* [176].

The method of applying microalgae-based biostimulants—whether through foliar spraying or root applications (soil fertilization or hydroponics)—can lead to different outcomes. For instance, the root application of *Spirulina platensis* to papaya (*Carica papaya*) seedlings demonstrated a more pronounced positive effect on plant growth and biomass production compared to foliar spraying [118]. Conversely, the foliar application of microalgae has been shown to significantly benefit plant growth in crops such as eggplant (*Solanum melongena*) [119], petunia (*Petunia x hybrida*) [120], garlic (*Allium sativum*) [121], and bell pepper (*Capsicum annuum*) [122] (Table 1). In addition, it was also reported that the foliar spray of *Nannochloris* sp. extracts induces drought stress tolerance in tomato (*Solanum lycopersicum)* [117].

The chemical composition of microalgae extracts displays both intra- and interspecific variability [134], with algal metabolomes undergoing significant alterations in response to stress conditions [177]. Among the key carbohydrates found in microalgae are polysaccharides such as β-glucan, which have been associated with notable biostimulant effects [178,179]. These polysaccharides interact with membrane receptors that regulate genes involved in cell expansion [180,181,182]. Microalgae also provide critical amino acids for plant metabolism, including tryptophan, arginine, proline, and glycine. These amino acids are essential for plant growth and development, functioning as precursors of phytohormones; for instance, tryptophan is indispensable for synthesizing indoleacetic acid, while arginine is vital for polyamine synthesis [183]. Furthermore, microalgae are also rich in betaines, vitamins, essential macro- and micronutrients, polyamines, and pigments (i.e., chlorophylls, carotenoids, and phycobilins). Carotenoids, in particular, serve as antioxidants that inactivate reactive oxygen species (ROS) generated by the exposure of cells to UV-B irradiation or stressful nutrient conditions [135,176,184,185,186,187]. Microalgae are also known to produce a wide range of plant hormones, such as auxins, cytokinins, gibberellins, ethylene, abscisic acid, and brassinosteroids [41,120,188,189,190,191,192,193]. Additionally, the content of essential macro- and micronutrients for plants indicates that microalgae-derived products could play a beneficial role as a slow-release fertilizer. In addition, metabolites identified in crude microalgae extracts, such as proline, glycine betaine, and polyphenols, play an important role in osmotic adjustment under salt stress and help protect cells from ROS [194].

Microalgae have the potential to revolutionize sustainable agriculture. Their ability to produce biomass efficiently, high nutrient content, and diverse range of bioactive compounds make them a promising source of plant biostimulants.

## 6. Bacteria-Derived Plant Biostimulant

Bacterial-based plant biostimulants include beneficial organisms such as plant growth-promoting rhizobacteria (PGPR) and phosphate-solubilizing bacteria (PSB). These kinds of biostimulants can improve plant growth and health under harsh environments [195,196]. Bacterial-based biostimulants work through different mechanisms, such as plant defense activation, enhancing nutrient uptake, and improving water use efficiency, among other processes. They can also stimulate plant tolerance to stress, including drought, salinity, and extreme temperatures.

PGPR comprise a set of naturally occurring rhizosphere bacteria, which are crucial in shaping soil–plant interactions [197]. In addition, they are host-specific and can be found freely in the rhizosphere, thus establishing epiphytic relationships with plants or engaging in symbiosis with roots, forming nodules [198,199,200]. A variety of bacterial genera can be found among soil PGPR, such as *Rhizobium*, which commonly establish nodules on legume roots [201]. PGPR are able to provide a variety of benefits to plants [195,196]. For instance, they enhance nutrient uptake, promoting the solubilization of insoluble nutrients, such as phosphate and iron, making them more readily available to plants. They can also produce plant growth regulators, which stimulate root growth and nutrient uptake. This increment in root biomass also improves water extraction from the soil, enhancing plant water use efficiency. In addition, PGPR can trigger signaling pathways involved in plant resistance to abiotic stresses, such as drought and salinity. On the other hand, PGPR suppress soilborne pathogens, for instance, competing for food and space or producing antibiotics [202]. In addition, they have showed the capacity to improve crop nutrient use efficiency, enhancing the availability of nutrients such as nitrogen, phosphorus, and potassium [198]. For instance, atmospheric nitrogen fixation is generally carried out by both symbiotic and free-living bacteria belonging to the genera *Bradyrhizobium*, *Rhizobium*, *Frankia*, *Mesorhizobium,* and *Sinorhizobium* [203,204]. Moreover, rhizobacteria promote the synthesis of growth-associated hormones, such as auxins, gibberellins, cytokinins, and abscisic acid, in response to stressful situations [205,206].

PGPR provide diverse mechanisms to induce abiotic stress tolerance, such as the biosynthesis of organic acids, exopolysaccharides, siderophores, and osmolytes (i.e., proline or betaine glycine), as well as the regulation of gene expression associated with immune responses [123,206,207,208]. On the other hand, when faced with an increase in the endogenous ethylene level induced by unfavorable environmental conditions, PGPR possess the ability to decrease the amount of this hormone by synthesizing the enzyme ACC (1-aminocyclopropane-1-carboxylic acid) deaminase since ACC is the precursor of ethylene [124,209,210]. Additionally, PGPR play a crucial role in enhancing the ROS scavenging system during abiotic stress conditions, leading to a reduction in oxidative damage. This is accomplished by synthesizing degradative enzymes such as catalases, peroxidases, and superoxide, along with the contribution of non-enzymatic antioxidants (i.e., phenolic compounds) [44,211,212,213]. In this sense, PGPR have been shown to enhance plant stress tolerance on different crops, such as amaranth (*Amaranthus viridis*) [125], barley (*Hordeum vulgare*) [126], maize (*Zea mays*) [127], rice (*Oryza sativa*) [124], date palm (*Phoenix dactylifera*) [123], and tomato (*Solanum lycopersicon*) [128], among others (Table 1).

On the other hand, phosphate-solubilizing bacteria (PSB) are able to significantly improve crop yield by increasing phosphorus (Pi) availability in soil. Pi is an essential nutrient for plant growth, but it is often the limiting factor in plant productivity [214,215]. PSB are a subgroup of PGPR that belong to the phyla *Proteobacteria*, *Firmicutes,* and *Actinobacteria*; among the best-known genera are *Rhizobium*, *Burkholderia*, *Pseudomonas*, *Enterobacter,* and *Bacillus* [216,217,218,219]. Their activity and distribution are influenced by soil type, natural microbiome, environmental and ecological conditions, and agronomic soil management [219,220]. These microorganisms can be used as biostimulants to replace chemical phosphate fertilizers since they can solubilize phosphate from both organic and inorganic sources [94,96,221]. Thus, substrate degradation and the enzymatic activity of phytases, non-specific phosphatases, and carbon–phosphorous lyases enable the biochemical and biological mineralization of organic Pi. On the other hand, the synthesis of siderophores, exopolysaccharides, and H_2_S enables the solubilization of inorganic Pi [99,222,223]. Likewise, medium acidification through the secretion of organic acids and chelation are mechanisms used by bacteria for Pi solubilization [214]. PSB also exhibit the ability to produce the growth regulator auxin and modify root architecture, thereby promoting plant growth and development [215]. In addition, these microorganisms also contribute to improving immunity against pathogens and the availability of some micronutrients.

Recent research has shown that the use of PSB enhances phosphorus acquisition and distribution in the plant, improving yield [223]. These results have been reported in crops such as maize (*Zea mays*) [224], oak (*Quercus Brantii*) [129], peanut (*Arachis hypogaea*) [130], potato (*Solanum tuberosum*) [131], and tomato (*Lycopersicon esculentum*) [132] (Table 1). Moreover, it has been reported that PSB not only increase Pi solubilization but also improve trace element availability and increase nitrogen use efficiency [133,220]. However, it should be noted that the use of PSB as a biostimulant is limited because only some of these bacteria are able to adapt to different agroecological conditions, and their pathogenic capabilities remain to be elucidated [223,225,226]. Despite this limitation, the potential of PSB to improve soil fertility and crop productivity is immense.

Bacterial-based biostimulants are a promising new approach to sustainable agriculture [227,228]. They offer benefits in terms of ecological crop production, given that microorganisms are generally considered to be more environmentally friendly than chemical inputs. Moreover, this kind of biostimulants can be specifically targeted to the needs of individual crops, soil conditions, or ecosystem properties. The use of microorganisms as plant biostimulants is still an evolving field of research. Further research is needed to optimize the formulation and application of microorganism-based biostimulants. However, the potential benefits of these biostimulants are significant, and they are likely to play an increasingly important role in sustainable and efficient agricultural system developments due to their reduced cost, environmental friendliness, and high efficiency.

## 7. Conclusions

In light of the increasing challenges posed by abiotic stressors and the urgent need for sustainable agriculture solutions, biostimulants are emerging as a powerful tool for enhancing crop resilience and productivity. These versatile substances, encompassing a wide variety of organic compounds and microbial formulations, offer a diverse array of applications that contribute to agricultural sustainability. Biostimulants play a pivotal role in hormonal regulation, antioxidant defenses, and metabolic adjustments, thereby exerting a multifaceted influence on plant physiology. Whether addressing drought, salinity, extreme temperatures, or nutrient deficiencies, biostimulants demonstrate remarkable potential to ameliorate these stressors and promote sustainable crop production. To fully harness their potential, a holistic and interdisciplinary approach to the optimization of biostimulants is essential. This includes tailoring formulations to specific crops and environmental conditions, integrating their use into precision farming practices, and developing innovative application methods. Continued research and technological advancements will be instrumental in realizing the full promise of biostimulants as key components of resilient and environmentally sustainable agricultural systems. As we look to the future, biostimulants are positioned to play a transformative role in shaping the next generation of global agricultural practices.

## 8. Future Perspectives: Optimizing Biostimulants for Sustainable Crop Production

The future of biostimulant research and application lies in their seamless integration into precision agriculture practices. By leveraging advanced technologies such as remote sensing, drones, and data analytics, it is possible to enable targeted and site-specific applications of biostimulants. This approach not only optimizes resource utilization but also enhances the efficacy of these substances while minimizing environmental impact.

As our understanding of the underlying mechanisms of biostimulant mechanisms action continues to evolve, new opportunities are emerging to tailor formulations to the specific requirements of different crops and environmental contexts. Customized biostimulant blends, combining synergistic organic and microbial components, have the potential to maximize their benefits and address unique challenges faced by diverse agricultural systems. However, despite the recognized potential of biostimulants, they remain a “black box” in terms of fully understanding the complexities of their mechanisms of action. Future research must focus on elucidating the precise mechanisms by which these biostimulants enhance plant performance, thereby enabling the development of more targeted and effective formulations.

Currently, establishing clear regulatory frameworks and standardized testing protocols for biostimulants is essential to ensure product quality, efficacy, and environmental safety. Collaborative efforts between researchers, industry stakeholders, and regulatory bodies are essential to create guidelines that encourage innovation while safeguarding the interests of farmers and the environment. Finally, the successful adoption of biostimulants in agriculture also hinges on widespread education and knowledge transfer. Outreach programs, training initiatives, and collaborative platforms bridging researchers and farmers will facilitate the dissemination of information about the benefits, application techniques, and best practices for biostimulant use. By addressing these challenges and fostering collaboration across disciplines, biostimulants can fulfill their potential as transformative tools for a sustainable agricultural future.
